# An Unusual Cervical Mass in the Hyoid Bone: Intermediate-Grade Chondrosarcoma

**DOI:** 10.1155/2019/7170832

**Published:** 2019-02-17

**Authors:** Ozge Caglar, O. Karatag, A. Avci, Sefa Dereköy

**Affiliations:** ^1^18 March University, Medical School, Otorhinolaryngology Department, Çanakkale, Turkey; ^2^18 March University, Medical School, Radiology Department, Çanakkale, Turkey; ^3^Izmir Katip Celebi University, Ataturk Education Hospital, Pathology Department, İzmir, Turkey

## Abstract

We describe a case of a 31-year-old woman with a chondrosarcoma of the hyoid bone. The patient presented with a mass in the left submandibular region. Fine-needle aspiration cytology suggested chondroma, but further imaging investigation with CT revealed an exophytic tumor originating from the body of the hyoid bone. Histopathology of the surgical specimen confirmed the diagnosis of a intermediate-grade chondrosarcoma. Chondrosarcomas account for 11% of all bone cancers. Primary sites of the head and the neck include the nasal cavity, the skull base, the maxilla, the mandible. Chondrosarcomas of the hyoid bone are very rare, with only 23 cases previously reported in the literature.

## 1. Introduction

Chondrosarcoma is a malignant tumor characterized by the formation of chondrogenic cartilage matrix [1]. These types of neoplasms are more common in the elongated bones, pelvis, and ribs. The ratio of chondrosarcoma found in the head and neck is very low, only 1% [1]. Chondrosarcomas of the hyoid bone are a very rare entity, and only 23 cases have been reported in the literature. The only treatment in these cases is surgery which is performed by removing the hyoid bone and providing a safe margin. Other tumors that can be seen in the hyoid include plasmacytomas, osteosarcoma, giant cell tumor, aneurysmal bone cyst, and benign osteoma. Patients with hyoid bone tumors usually present with dysphagia or palpable neck masses.

In this case report, we will discuss a patient who was referred for surgery with hyoid chondrosarcoma.

## 2. Case Report

**Figure 1:**
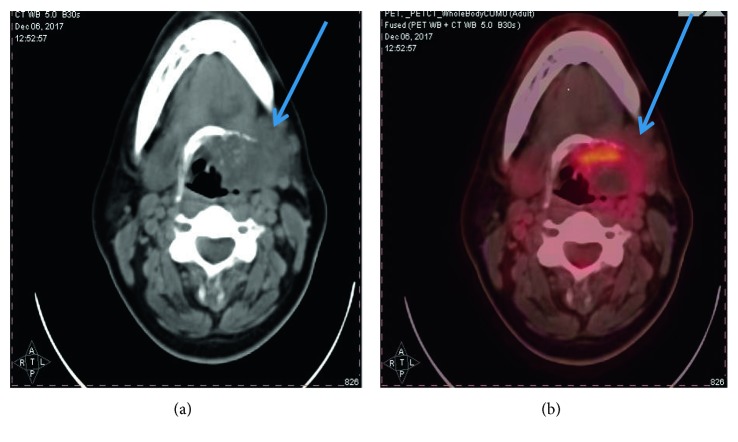
PET scan.

Permission was taken for publication from the patient. A 31-year-old female patient was admitted due to a mass in the left submandibular region in 2015, and mass excision surgery was performed. The result was reported as pleomorphic adenoma. Two years later, the patient was again referred with a mass in the same region. Fine-needle aspiration biopsy accompanied by ultrasonography was performed, and the result was reported as chondroma. Neck exploration was performed, and the mass was excised from the neck. During the operation, it appeared that the mass was very hard and could only be removed from the mylohyoid bone with sharp dissection. Pathology was reported as a low-grade chondrosarcoma. On control PET, which was performed for the patient again due to mass complaints, there was a mass with left submandibular gland localization, invading the left lateral wall of the larynx and destroying the left side of the hyoid bone (Figure 1).

**Figure 2:**
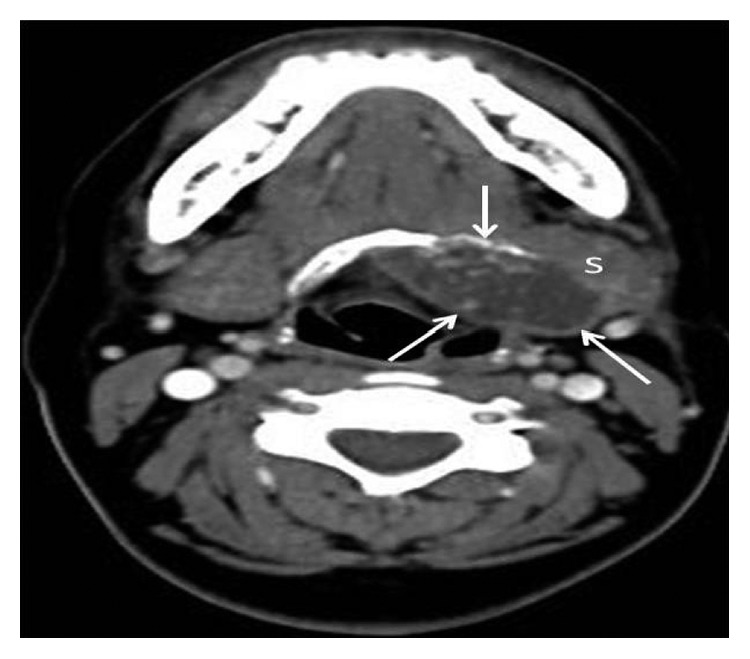
Contrast-enhanced axial (CT) image: expansile mass originating from the left side of the hyoid bone with cystic-necrotic components and calcific foci (arrows). The mass produces compressive effect on the left submandibular gland (S) and vallecula.

On neck CT scan, a mass of 4.5 × 2.5 cm, which was considered to have hyoid bone origin, was localized on the left side of the hyoid bone (Figure 2).

**Figure 3:**
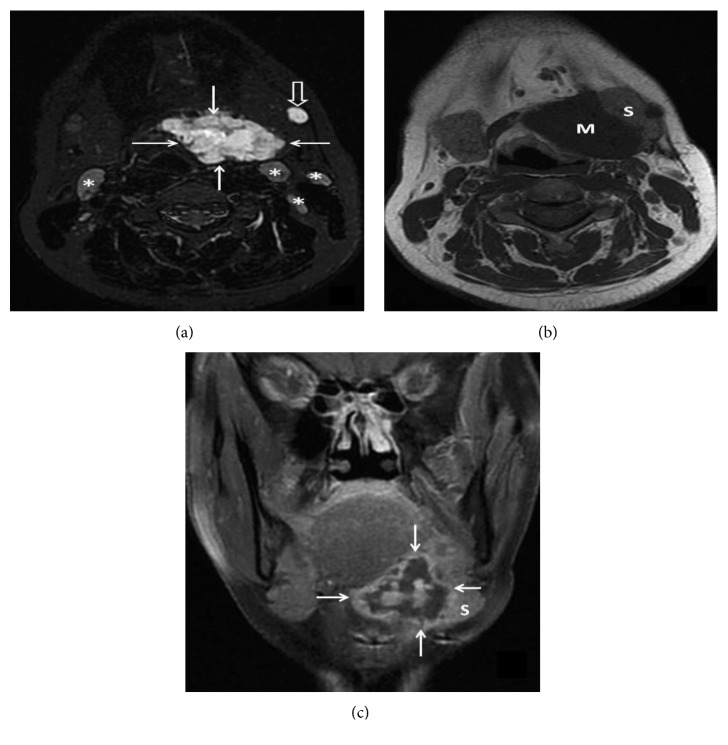
(a) Axial fat-sat T2 MRI image shows marked hyperintense mass with lobulated contours (arrows). There are metastatic lymph nodes within the left submandibular area (open arrow) and both sides of the neck lymph node chain (asterisk) with similar signal characteristics. (b) Axial T1 MRI image demonstrates effacement of the fat planes between the mass (M) and the left submandibular gland (S). (c) Coronal contrast-enhanced fat-sat T1 image reveals that the mass has an heterogeneous peripheral and central enhancement with some cystic-necrotic components (arrows).

When MRI images are taken, the metastatic lymph node nodules are seen (Figures 3(a)–3(c)).

With these results, left supraomohyoid neck dissection and hyoid resection of the tumor were performed on the patient.

**Figure 4:**
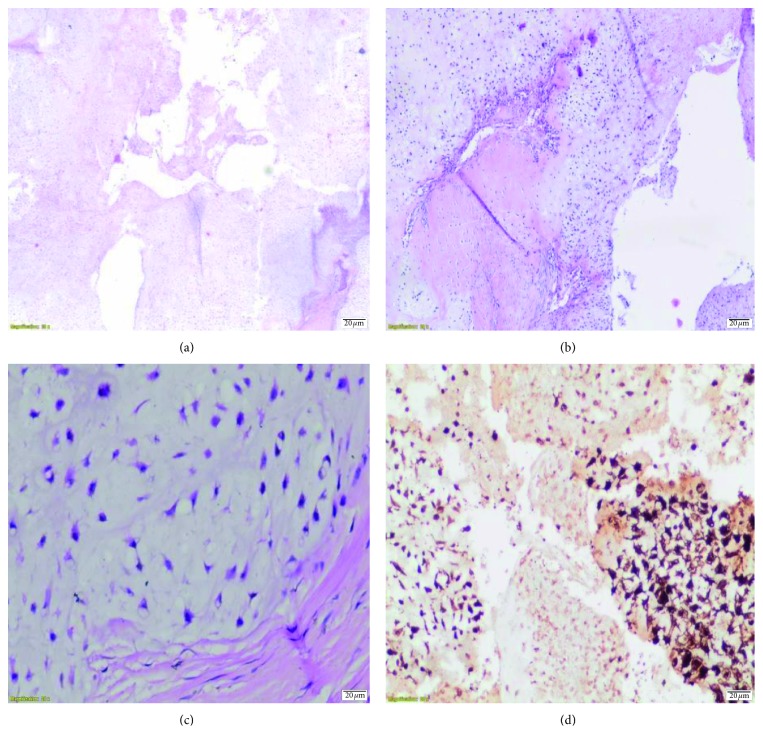
(a) Nodular chondroid fields, ×2, H&E. (b) Nodular chondroid areas, stellate and lacunae, atypical, pleomorphic cells, ×10, H&E. (c) Pleomorphic hyperchromatic, grade II, ×40, H&E. (d) Tumor cells in s100 immunohistochemical study.

Histologically, chondrosarcomas, cell atypia, and cellularity are divided into three subgroups according to their characteristics. Myxoid change is frequently observed. This is considered to be intermediate if there is a mycoid structure even if the cellularity is low (Figures 4(a)–4(c)). Pathology was reported as an intermediate-grade chondrosarcoma (Figure 4(d)).

During the postoperative period, the patient received radiotherapy treatment. No recurrence was observed after radiotherapy. The patient is currently seen once a year.

## 3. Discussion

Mesenchymal chondrosarcomas originating from cartilage matrix cells are divided into primary and secondary types [2]. Primary chondrosarcomas are unusual and occur in the central region of the bones, especially in children. Secondary chondrosarcomas develop in previously existing benign lesions such as osteochondromas or enchondromas. It is common in the 30–60 age group [3], reaching a peak around 40 years.

No significant gender difference was found in the incidence of chondrosarcoma [3]. Chondrosarcomas mostly develop in the skeletal area: pelvis, femur, proximal humerus, or rib bones [4]. It is a rare disease in the head and neck region and represents about 0.1% of developing malignancies in these regions [5].

Common areas for chondrosarcoma in the head and neck are skull base, nasal cavity, maxillary bone, mandible, and larynx. Hyoid chondrosarcomas are extremely rare tumors, and there are about 23 cases reported previously in the literature [6]. Hyoid chondrosarcomas may also be seen as painless, asymptomatic, and palpable masses, including symptoms related to respiration and symptoms such as voice anxiety and dysphagia. In our case, the patient was referred to our clinic with complaint about a mass.

The hyoid chondrosarcoma in the literature is mainly grade 1, with only 4 cases of grade 2 chondrosarcoma [6]. Interestingly in our case, the specimen from the first operation of the patient was reported as grade 1 chondrosarcoma and grade 2 was reported after the last operation.

Chondrosarcomas tend to be lobular masses that enlarge and alter the morphology of the primitive region. These are characterized by the formation of hyaline cartilage, and the cross-sectional surfaces are hardened by calcification [7]. In our case, the specimen was rigid in both surgeries, requiring sharp dissection for excision.

The majority of chondrosarcomas are of low grade, and the metastatic rate is low. Metastatic potential peaks are only around 5% and spread to the lung and skeletal system [8]. In order to be able to circumvent these conditions, we also performed PET on our patient.

CT imaging shows lobular, destructive, heterogeneous soft tissue mass and is associated with matrix calcification in 75% of cases [9].

CT is the best imaging method to show osteoclasis or cortical erosion, which is usually associated with such tumors as well as the characteristic calcification pattern.

MR helps to identify the soft tissue size of the lesion and thus helps in planning the surgical procedure [9]. Chondrosarcomas characteristically provide a high signal on T2-weighted images and a low-to-medium signal on T1-weighted images.

Cytopathology of these suprahyoid neck masses can lead to misdiagnoses such as pleomorphic adenomas originating from the submandibular or sublingual glands. For this reason, imaging of these lesions before surgery is very important. In addition, unlike other common areas of the head and neck, hyoid bone chondrosarcomas tend to occur as painless, generally slow-growing palpable masses, with symptoms, lack of evidence, and nonaggressive behavior indicating benign masses which may lead to a more protective surgical approach. However, the recommended treatment is extensive local surgical excision with total hyoidectomy to reduce the chance of recurrence and histologic differentiation. In our case, the first surgeon reported that the tongue tissue was the source of the tumor, and it was reported as pleomorphic adenoma. The second surgery at the hospital was reported as low grade, and an intermediate-grade chondrosarcoma was reported after the last operation. This shows how pathology may be inadequate and how important it is to visualize the disease.

Histopathologic diagnosis of the degree of chondrosarcoma is sometimes very difficult. This is worrisome because the treatment of first-degree chondrosarcomas and second-degree chondrosarcomas is different. Therefore, it is essential to develop new histopathological diagnostic methods to increase diagnostic accuracy (molecular medicine, immunohistochemistry, and cytogenetics) [10].

When the average age of the cases in the literature is examined, it changes between 40 and 69 years while our patient is a young 31-year-old woman. This shows that these tumors have begun to increase in young patients.

The most important treatment is surgery of the hyoid bone. Neither radiotherapy nor chemotherapy plays an important role in primary treatment. Chondrosarcoma is considered to be radiation resistant. For high-grade tumors, partially resected tumors, and patients who do not accept surgery or palliative treatment, adjuvant radiotherapy may be recommended [11]. In the literature, the treatment applied to the illness is usually decided by the surgeon, and the use of RT is appropriate for cases rejecting the surgery or extending to the base of the head. In 6 cases, total hyoid bone resection was applied, and in 10 cases, only large corn and abscesses were removed [6]. Additional treatment, surgical excision adequacy, tumor location, and tumor histological grade are the most important prognostic factors. The possibility of metastasis correlates with poor prognosis-inducing malignancy grade [12]. Five-year survival rates for all chondrosarcoma cases were 90% for first grade, 81% for second grade, and 43% for third grade [13]. For this reason, periodic monitoring is necessary. In addition, local regional recurrences should be investigated. Chest radiography is recommended every six months because lung metastasis is the most common form of metastasis.

In conclusion, a very rare condition and treatment is desired to be discussed here. Therefore, if there is a mass in this region in the patients who have been referred to the clinic, evaluation of the hyoid bone is suggested.
